# Immunosuppression Reversal Nanovaccines Substituting Dendritic Cells for Personalized Cancer Immunotherapy

**DOI:** 10.3389/fimmu.2022.934259

**Published:** 2022-06-24

**Authors:** Hu Chen, Hongwei Cheng, Xiaoliu Liang, Shundong Cai, Gang Liu

**Affiliations:** State Key Laboratory of Molecular Vaccinology and Molecular Diagnostics and Center for Molecular Imaging and Translational Medicine, School of Public Health, Xiamen University, Xiamen, China

**Keywords:** nanovaccines, antigen self-presentation, immunosuppression reversal, cancer immunotherapy, dendritic cells

## Abstract

Although immunotherapy has paved a new avenue for cancer treatment, inadequate immune response often executes suboptimal therapeutic effects. In general, an effective immune response undergoes presentation of antigen by antigen-presenting cells, proliferation and differentiation of lymphocytes, and attack of cancer cells by cytotoxic T lymphocytes (CTLs). The antigen self-presentation and immunosuppression reversal (ASPIRE) nanovaccine derived from dendritic cells provides a simplified and immune deregulated procedure for immunotherapy profiting from its orientable peculiarity. By integrating major histocompatibility complex class I (MHC-I) molecules into present specific epitopes and co-delivering anti-PD-1 antibody and B7 costimulatory molecules through the programmed biomimetic synthesis, the ASPIRE nanovaccine demonstrates a milestone in personalized cancer immunotherapy.

## Introduction

In recent years, substantial progress has been made in cancer immunotherapy, especially the clinical application of immune checkpoint antibody and the chimeric antigen receptor T-cell (CAR-T), which has brought new light to cancer treatment. Although current immunotherapy is effective in the treatment of hematological cancers, the immune efficiency against solid tumor is frustrating (1). The main reasons are inefficient immune responses and an immunosuppressive tumor microenvironment. Theoretically, the anti-tumor immunological process involves several consecutive steps, including the presentation of antigen by antigen-presenting cells (APCs), the proliferation and differentiation of lymphocytes, and the attack of cancer cells by cytotoxic T lymphocytes (CTLs). However, this progressive procedure is likely affected by numerous factors, such as the efficiency of maturation, differentiation, and migration of dendritic cells (DCs); the transmission of antigen signal to T lymphocytes from DCs; the differentiation of T lymphocytes; and the invasion of T lymphocytes into the tumor environment. Among all these factors, the recognition and presentation of antigens by DCs is the first and considered to be the most pivotal step (2). Thus, the development of vaccine is typically focused on stimulating DCs to facilitate their maturation, proliferation, and differentiation. Even so, the increment of CTLs that play a role in killing tumor cells is actually not significantly ameliorated (3). In addition, a tumor immunosuppressive microenvironment is another important factor that restricts anti-tumor immunity, which should be considered. For example, immune checkpoints can inhibit the activity of immune cells by leading to the exhaustion of T cells (4). In order to overcome the above obstacles and amplify the anti-tumor immune efficiency, simplifying the above response procedures together with reversing the immunosuppressive environment may be an efficient resolution strategy.

Recently, the development of nanotechnology has greatly expanded therapy strategies for enhancing anti-tumor immunity (5). In particular, studies indicate that nanoparticles as nanovaccines or drug carriers exhibit faculty for reshaping the tumor microenvironment and regulating the forceful immune response (6). The membrane nanovesicle (NV), which is commonly extracted from engineered eukaryotic cells expressing interesting protein or peptide, maintains the natural conformation, structure, and activity of the function, and is therefore considered as an ideal platform for biopharmacy (7). Recently, engineered nanovaccines based on NVs were fabricated for boosting the ability to stimulate immune responses and regulating the tumor immunosuppressive microenvironment (8). Remarkably, the antigen self-presentation and immunosuppression reversal (ASPIRE) nanovaccine fabricated by Liu et al. substituted dendritic cells for personalized cancer immunotherapy (9). ASPIRE is one of the NVs with an inherent ability of antigen presentation and was developed from the edited DCs, but the immune effect was better than that of DCs, and could even replace DCs to participate in the immune response. This excellent ability enables it to directly interact with T cells for antigen self-presentation, which can obviously improve the efficiency of immune response. Furthermore, anti-PD-1 antibody displayed on ASPIRE together with the original membrane protein on ASPIRE can antagonize immune checkpoints and achieve immunosuppression reversal, which can enhance the anti-tumor immune efficacy significantly. More importantly, antigen signal presented by ASPIRE was tailored according to tumor neoantigens, which can be widely used in personalized immunotherapy for various types of cancer.

## ASPIRE Nanovaccine for Personalized Cancer Immunotherapy

**Figure 1:**
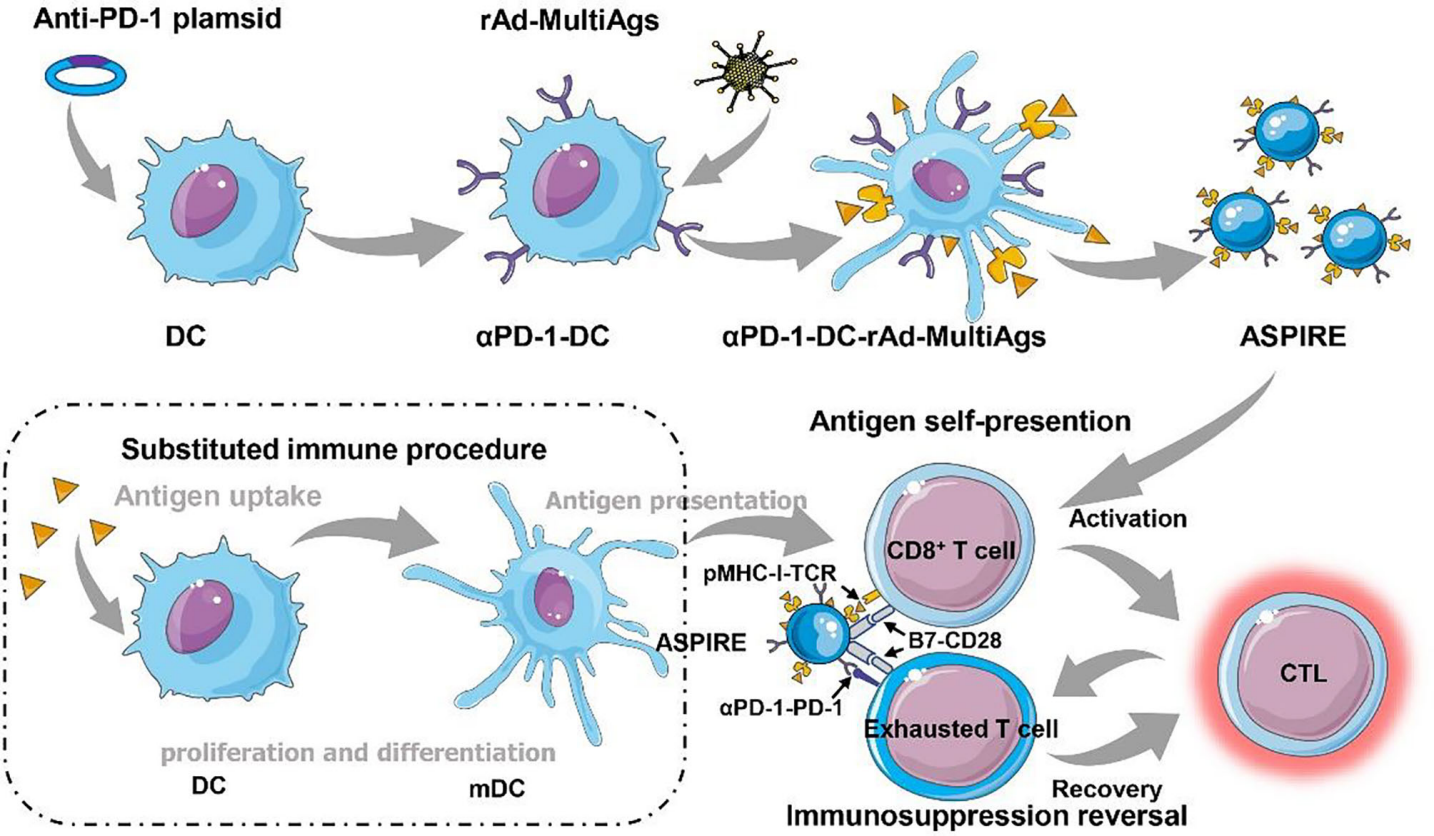
Immunosuppression reversal nanovaccines (ASPIRE) substituting dendritic cells for personalized cancer immunotherapy. The ASPIRE nanovaccine was fabricated *via* integrating anti-PD-1 antibody, neoantigen, specific peptide-major histocompatibility complex class I (MHC-I), and B7 costimulatory molecules into a single bionic nanosystem through the directional expression technique, which exhibited the capabilities of antigen self-presentation and immunosuppression reversal substituting DC-dependent immune procedure.

ASPIRE was prepared *via* integrating anti-PD-1 antibody, neoantigen, specific peptide-major histocompatibility complex class I (MHC-I), and B7 costimulatory molecules into a single bionic nanosystem through the directional expression technique (**Figure 1**). The ASPIRE nanovaccine exhibited efficient enrichment in the lymph node system due to its nanoscale size, good stability, and targeting effect mediated by the surface adhesion molecule. Deriving from DCs, ASPIRE possesses the capacity of antigen presentation, imitating DCs to act as a signal transducer to T lymphocytes. This procedure is termed “antigen self-presentation”, which demonstrated that ASPIRE could significantly improve the efficiency of the immune response. Meanwhile, due to the presence of the CD28/B7 costimulatory molecule, the original inhabitant of the NVs, the checkpoint inhibition ability of anti-PD-1 antibody is elevated, promoting the reversal of immunosuppression to execute a long-term anti-tumor immune response. This powerful vaccine formula directly activates both native T cells and exhausted T cells, demonstrating a marvelous strategy for personalized cancer immunotherapy. This engineered nanovaccine with both antigenic self-presentation and immunosuppression reversal capabilities is defined as ASPIRE. In this part, how ASPIRE plays the function of antigen self-presentation and immunosuppression reversal will be introduced and discussed briefly.

Immune strategies that directly act on T lymphocytes have opened the way for simplifying immune response procedures. Model antigens were anchored on the cytomembrane of mature DCs differentiated from immature DC2.4 cells through recombinant adenovirus, while the upregulation of the expression of MHC-I on DCs was induced. DC nanovesicles (DCNVs) engineered by recombinant adenovirus expressing membrane localization modified antigen (rAd-Ag) were harvested *via* multistep density gradient ultracentrifugation. In particular, costimulatory molecules and various chemokines were confirmed to be overexpressed on DCNV-rAd-Ag, which contributed to the process of immune response, such as antigen presentation and migration to lymph nodes (10). When naive CD8^+^ T cells encounter the DCNV-rAd-Ags, specific proliferation and activation of CD8^+^ T cells were dramatically elicited, illustrating that DCNV-rAd-Ags can excite T cells straightforwardly. The merit of DCNV-rAd-Ags that are capable of antigen self-presentation derives from the inheritance of the complete surface functional proteins on mature DCs. Moreover, DCNV-rAd-Ags presented a better tropism effect on lymph nodes than whole DCs, attributed to lymphoid homing molecules from mature DCs and structural characteristics of itself. Relying on the CD28/B7 costimulatory molecule *in vivo*, robust CD8^+^ T-cell responses were elicited by DCNV-rAd-Ags, while this process was independent of DCs. Different from conventional vaccines, DCNV-rAd-Ags contain the original proteins on DC membrane and have antigen self-presentation capacity, which is similar to but more effective than DCs. The nano-bionic system provokes a strong specific immune response overlapping with the DC-mediated procedure, which removes the restriction of DC participation and significantly improves the efficiency of anti-tumor immunity.

ASPIRE was further devised based on engineered DCs incorporating αPD-1 and rAd-MultiAgs (αPD-1-DCNV-rAd-MultiAgs) for disrupting the immunosuppressive PD-1/PD-L1 pathway. Generally, the anti-tumor immune effect is restricted, mainly because the T cells are imprisoned by the tumor immunosuppressive environment. The existence of immune checkpoint is deemed to be one of the main nuisances that hold back the aggressivity of T cells. The anti-PD-1 antibody expressed on ASPIRE by programmed editing can effectively interdict the PD-1/PD-L1 pathway. Accordingly, ASPIRE realized the reversal of the immunosuppressive environment and rescued the T cells blocked in the tumor region. Additionally, the B7-CD28 signaling pathway was found to be associated with the intervention of immune checkpoint (11). When ASPIRE was administered, the frequency of PD-1^+^CD38^hi^ in tumor-infiltrating CD8^+^ T cells decreased significantly while the frequency of tumor-infiltrating antigen-specific CD8^+^ T cells showed the opposite trend. As expected, inherent costimulatory molecules on ASPIRE further facilitated immunosuppression reversal mediated by anti-PD-1 antibody. It was confirmed that ASPIRE could overcome stubborn immune tolerance, eradicate tumor growth, and maintain a lasting CTL response in tumor-bearing mice. ASPIRE treatment resulted in 100% tumor suppression on the MC-38 ectopic transplanted tumor model, which was superior to the general combination therapy of αPD-1-DCNV+DCNV-rAd-neo or anti-PD-1+DCNV-rAd-neo.

## Discussion

ASPIRE showed excellent tumor inhibition in preclinical studies while exerting the prospect of clinical transformation. The key of ASPIRE in the actual application is screening and identifying corresponding tumor neoantigen, because only the transmission of an appropriate antigen signal can stimulate a specific anti-tumor immune effect. With the development of immunomics and biotechnology, the identification and sequence analysis of neoantigens are increasingly adapted to clinical needs, providing a favorable basis for the potential clinical application of personalized immunotherapy through an ASPIRE immunization strategy (12). It is also worth mentioning that the synthesis of membrane vesicles is capable of quality control in a standard operating procedure and that appropriate proportions have been established. Compared with cumbersome and expensive cell therapy, ASPIRE formulation based on cell membrane vesicles has the advantages of high yield, easy preservation, and low cost. In the future, cell-free biomimetic immune agents may shine in the clinical application of immunotherapy.

The research and development of new nanovaccine platforms based on the membrane nano-bionic system shows a broad application prospect in cancer immunotherapy. The new concept of antigen self-presentation in immunotherapy breaks the traditional immune response process and achieves efficient antigen presentation and T lymphocyte immune response. The costimulatory molecules on DCs enhance immune checkpoint inhibition, break immune tolerance, realize immune inhibition reversal, and enhance the anti-tumor immune effect. The ASPIRE nanovaccine paves a new avenue for immunotherapy and is a milestone in nanovaccine development. Moreover, ASPIRE is just one of the representative applications of targeted expression of membrane proteins in personalized cancer vaccine development, and the technology platform could be extended to the treatment of other diseases, such as bacterial infections and chronic viral infections (13).

## Author Contributions

GL and HuC wrote the first draft of the manuscript. All authors contributed to the article and approved the submitted version.

## Funding

This work was supported by the Major State Basic Research Development Program of China (No. 2017YFA0205201) and the National Natural Science Foundation of China (NSFC, Nos. 81925019 and U1705281).

## Conflict of Interest

The authors declare that the research was conducted in the absence of any commercial or financial relationships that could be construed as a potential conflict of interest.

## Publisher’s Note

All claims expressed in this article are solely those of the authors and do not necessarily represent those of their affiliated organizations, or those of the publisher, the editors and the reviewers. Any product that may be evaluated in this article, or claim that may be made by its manufacturer, is not guaranteed or endorsed by the publisher.
